# Clinical efficacy of eyelid hygiene in blepharitis and meibomian gland dysfunction after cataract surgery: a randomized controlled pilot trial

**DOI:** 10.1038/s41598-020-67888-5

**Published:** 2020-07-16

**Authors:** Youngsub Eom, Kyung Sun Na, Ho Sik Hwang, Kyong Jin Cho, Tae-Young Chung, Roo Min Jun, Byung Yi Ko, Yeoun Sook Chun, Hyun Seung Kim, Jong Suk Song

**Affiliations:** 1 Department of Ophthalmology, Korea University Ansan Hospital, Korea University College of Medicine, Gyeonggi-do, South Korea; 2 Department of Ophthalmology, Yeouido St. Mary’s Hospital, College of Medicine, The Catholic University of Korea, Seoul, South Korea; 3 Department of Ophthalmology, Dankook University College of Medicine, Cheonan, South Korea; 4 Department of Ophthalmology, Samsung Medical Center, Sungkyunkwan University School of Medicine, Seoul, South Korea; 5 Department of Ophthalmology, Ewha Woman’s University School of Medicine, Seoul, South Korea; 6 Department of Ophthalmology, Konyang University College of Medicine, Daejeon, Republic of Korea; 7 Department of Ophthalmology, Chung-Ang University Hospital, Chung-Ang University College of Medicine, Seoul, South Korea; 8 Department of Ophthalmology, Seoul St. Mary’s Hospital, College of Medicine, The Catholic University of Korea, Seoul, South Korea; 9 Department of Ophthalmology, Korea University Guro Hospital, Korea University College of Medicine, Seoul, South Korea; 10 Korean Meibomian gland & Ocular Surface study group, Seoul, South Korea

**Keywords:** Eye diseases, Medical research, Outcomes research, Clinical trial design

## Abstract

The purpose of this randomized clinical trial is to evaluate the effect of eyelid hygiene on subjective symptoms, anterior blepharitis, and meibomian gland dysfunction (MGD) after cataract surgery. Subjects with obstructive MGD who underwent cataract surgery were randomly divided into two groups. In the eyelid hygiene group, eyelid hygiene was performed twice a day for 10 days from 3 days before to 1 week after cataract surgery. The control group did not perform eyelid hygiene. A subjective symptom questionnaire of SPEED, anterior blepharitis grade, and meibum quality and quantity was evaluated at baseline and at postoperative 1 and 4 weeks. The eyelid hygiene group (n = 36) showed decreased SPEED score after cataract surgery and the control group (n = 33) did not. Anterior blepharitis grade was worse 1 week after surgery in the control group but not in the eyelid hygiene group. The control group had significantly decreased meibum quality and quantity in both the upper and lower eyelids after cataract surgery, but the eyelid hygiene group did not. Eyelid hygiene before/after cataract surgery improved postoperative subjective symptoms and prevented postoperative exacerbation of anterior blepharitis and MGD. Thus, perioperative eyelid hygiene is recommended for patients with obstructive MGD who undergo cataract surgery.

## Introduction

Blepharitis is divided into anterior and posterior blepharitis according to anatomic location. Meibomian gland dysfunction (MGD) is the main cause of posterior blepharitis. MGD is characterized by abnormal meibomian gland secretion, qualitative/quantitative changes in meibum, dry eye symptoms, and ocular surface inflammation^1–3^. Anterior blepharitis and MGD are important factors that may cause evaporative dry eye syndrome^4,5^. They cause deficiency or imbalance of the tear film lipid layer secreted from meibomian glands to increase tear evaporation even if aqueous tear secretion is normal^6,7^. On the other hand, another study reported that the tear film lipid layer itself may not inhibit the rate of tear evaporation^8^. Nevertheless, an important function of the tear film lipid layer is to spread tears to the ocular surface between blinks, helping to maintain tear film homeostasis on the ocular surface.

Previous studies have reported that the signs of blepharitis can worsen after cataract surgery, and the degree of dry eye syndrome and MGD is worse than before surgery^9–12^. More significant worsening of MGD was observed in patients with severe preoperative MGD compared to those with minimal preoperative MGD^10^. Thus, the American Society of Cataract and Refractive Surgery (ASCRS) Cornea Clinical Committee recently proposed an algorithm for preoperative diagnosis and treatment of ocular surface diseases, including MGD, to improve postoperative satisfaction and visual outcome by preoperatively treating ocular surface disease^13^.

Treatment of blepharitis and MGD includes eyelid hygiene, warm compress, and topical and systemic antibiotic therapy^14–17^. Warm compress is conducted to heat the eyelid above the phase transition temperature of meibum which results in more meibum delivery to the eyelid^18^. Eyelid hygiene is reported to be particularly effective in eliminating waste products such as crusts, waxy meibum, and bacterial lipase in the eyelids and improving symptoms of anterior and posterior blepharitis^19–22^. In addition, eyelid hygiene performed before ophthalmic surgery is reported to significantly reduce bacteria in the eyelids^23^. The purpose of this study was to evaluate the efficacy of eyelid hygiene, which is used as a standard treatment for blepharitis and MGD, before and after cataract surgery in preventing the development and worsening of blepharitis and MGD and to improve symptoms after cataract surgery.

## Results

**Figure 1:**
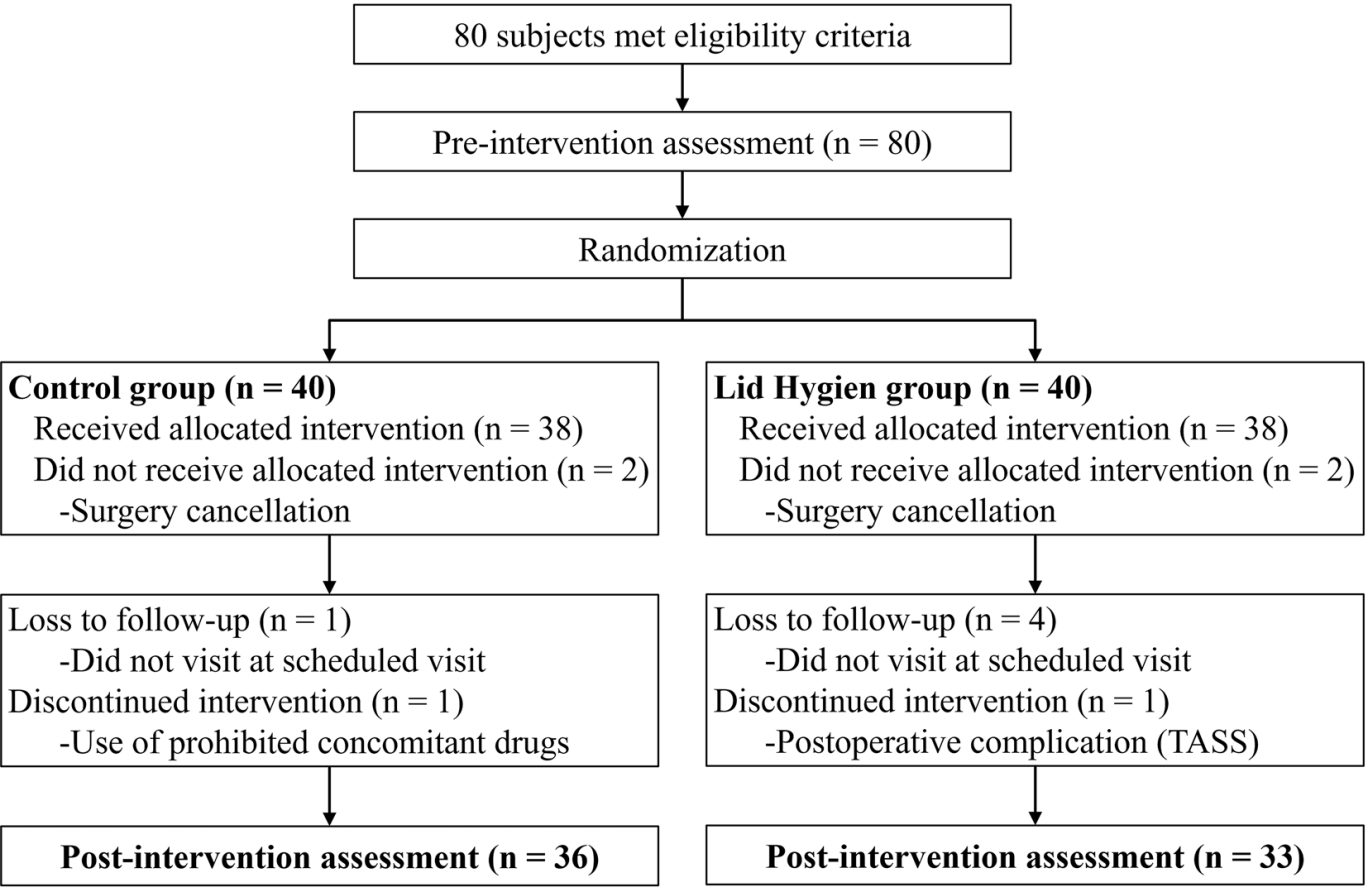
Flow diagram of a randomized controlled pilot trial on clinical efficacy of eyelid hygiene in blepharitis and meibomian gland cataract surgery. TASS = toxic anterior segment syndrome. Figure was created by Microsoft PowerPoint 2016.

A total of 69 eyes from 69 patients with cataract (of 80 prescreened and 80 enrolled) completed the clinal study protocol and pre- and post-operative assessments (33 eyes of 33 in the eyelid hygiene group and 36 eyes of 36 patients in the control group) (Fig. 1). The mean age of the control and eyelid hygiene groups were 66.3 ± 12.6 and 66.1 ± 9.7 years, respectively. There were 20 males (52.2%) in the control group and 17 males (45.9%) in the eyelid hygiene group. There were no significant differences in age and sex between the two groups.

**Figure 2:**
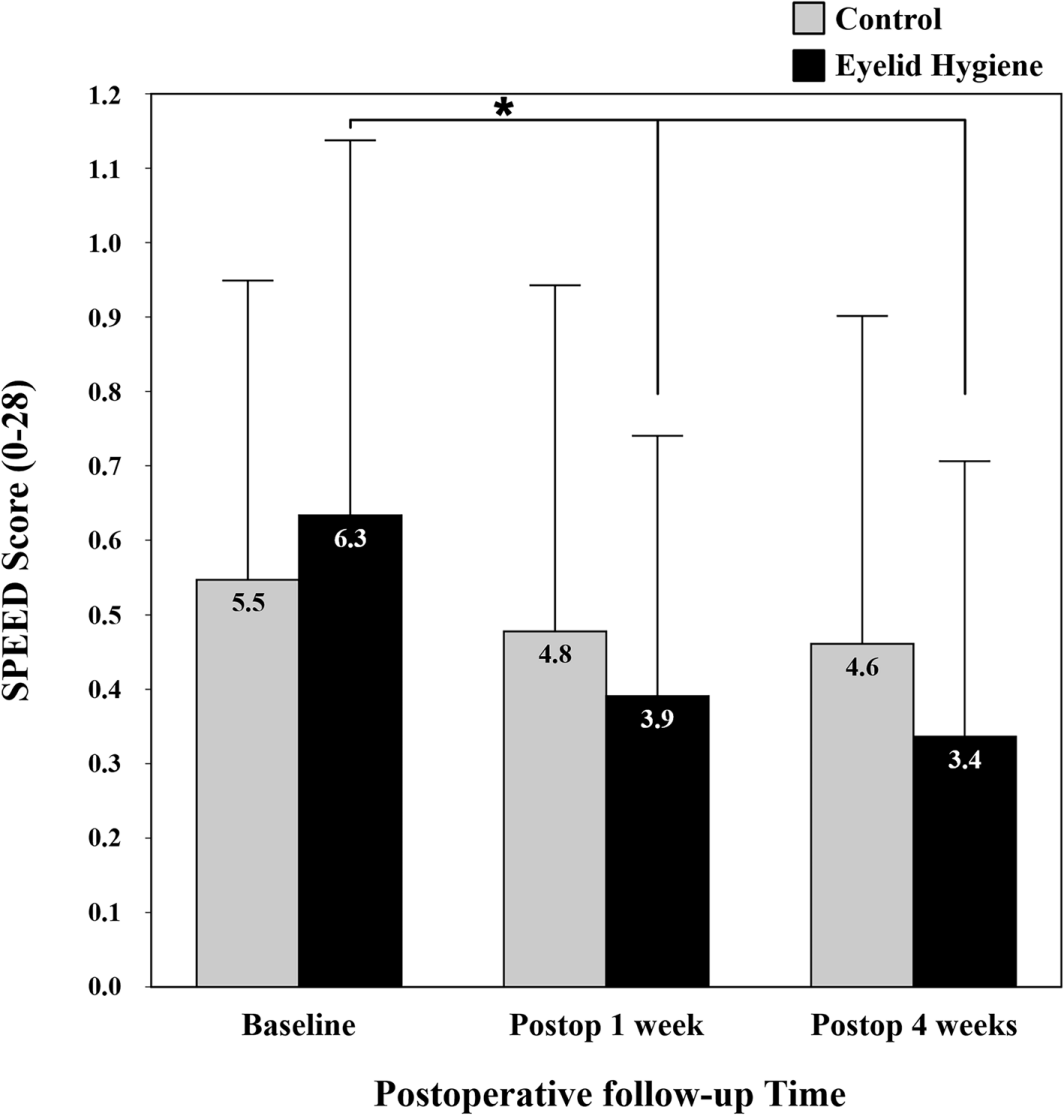
Comparison of subjective symptom scores between control and eyelid hygiene groups. The Standard Patient Evaluation of Eye Dryness Questionnaire (SPEED) was used to assess patient subjective symptoms and yields a score from 0 to 28. A score of 0 is the best score, and a score of 28 is the worst score. Asterisk indicates a *P* value < .05 by the repeated measures ANOVA with a Bonferroni correction. Figure was created by IBM SPSS Statistics Standard 20 and Microsoft PowerPoint 2016.

The eyelid hygiene group had a significant decrease in SPEED score (a score of 0 is the best score, and a score of 28 is the worst score) during the first 1 week after cataract surgery, which remained until postoperative 4 weeks. However, the control group showed no significant change in SPEED score during the same period (Fig. 2).

**Figure 3:**
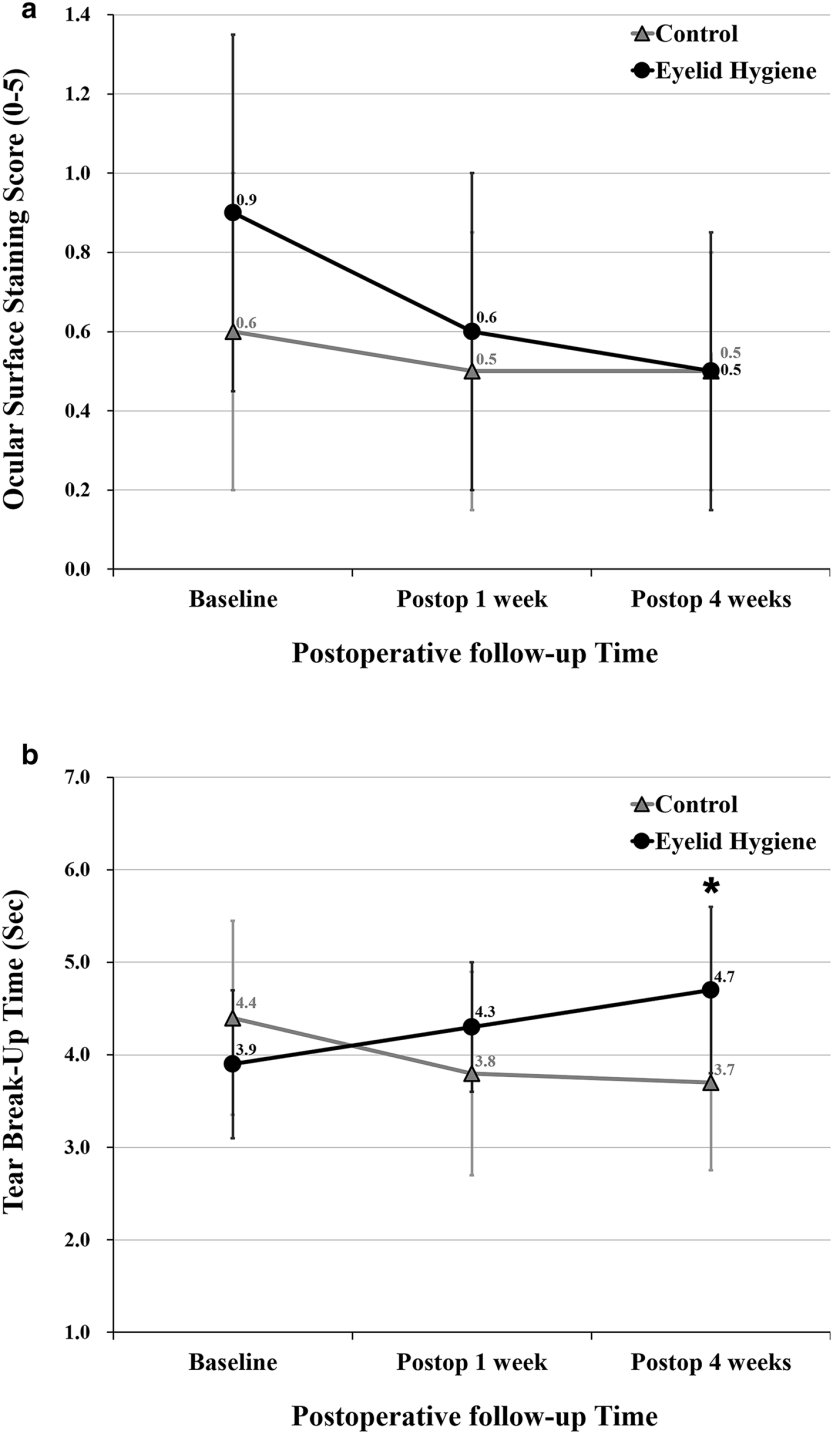
Comparison of ocular surface staining score and tear break-up time between control and eyelid hygiene groups. (**A**) Ocular surface staining score. (**B**) Tear break-up time. Asterisk indicates a *P* value < .05 by the repeated measures ANOVA with a Bonferroni correction. Figures were created by Microsoft PowerPoint and Excel 2016.

There were no significant changes in Oxford scale during the 4 week period after cataract surgery in both eyelid hygiene and control groups (Fig. 3A). TBUT tended to increase postoperatively in the eyelid hygiene group and decrease in the control group, although the difference was not statistically significant. As a result, mean TBUT was significantly higher in the eyelid hygiene group (4.7 ± 1.8 s) than in the control group (3.7 ± 1.9 s) at 4 weeks postoperatively (*P* = 0.030; Fig. 3B).

In evaluation of degree of anterior blepharitis, the mean grade of eyelid debris and telangiectasia increased significantly for 1 week immediately after surgery and then improved somewhat in the control group. Conversely, there was no worsening of the mean grade of eyelid debris and telangiectasia during the 4 week period after surgery in the eyelid hygiene group. The eyelid hygiene group had significant improvement in eyelid redness/swelling for 1 week immediately after surgery but the control group did not (Table 1).

**Table 1:** Comparison of anterior blepharitis and inflammatory signs between control and eyelid hygiene groups.

	Control (n = 36) (Mean ± SD)	Eyelid Hygiene (n = 33) (Mean ± SD)	*P* value*
Eyelid debris grade (0–4)			
Baseline	0.7 ± 0.7	0.8 ± 0.8	0.584
Postop 1 week	1.2 ± 0.9	0.6 ± 0.6	**0.002**
Postop 4 weeks	1.0 ± 0.9	0.6 ± 0.7	0.090
* P* value*			
Baseline vs. postop 1 week	**0.001**	0.547	
Baseline vs. postop 4 weeks	0.144	0.513	
Postop 1 week vs. 4 weeks	0.207	> 0.999	
Eyelid redness/swelling grade (0–4)			
Baseline	0.9 ± 0.6	0.9 ± 0.7	0.750
Postop 1 week	1.1 ± 0.8	0.6 ± 0.7	**0.010**
Postop 4 weeks	1.0 ± 0.7	0.7 ± 0.7	0.150
*P* value*			
Baseline vs. postop 1 week	0.146	**0.005**	
Baseline vs. postop 4 weeks	> 0.999	0.146	
Postop 1 week vs. 4 weeks	0.707	0.666	
Telangiectasia grade (0–3)			
Baseline	1.1 ± 0.7	1.1 ± 0.7	0.844
Postop 1 week	1.4 ± 0.9	1.0 ± 0.8	0.063
Postop 4 weeks	1.3 ± 0.8	1.1 ± 0.7	0.317
*P* value*			
Baseline vs. postop 1 week	**0.036**	> 0.999	
Baseline vs. postop 4 weeks	0.194	> 0.999	
Postop 1 week vs. 4 weeks	0.365	0.690	

*SD* standard deviation.

* Repeated measures ANOVA with Bonferroni correction.

**Figure 4:**
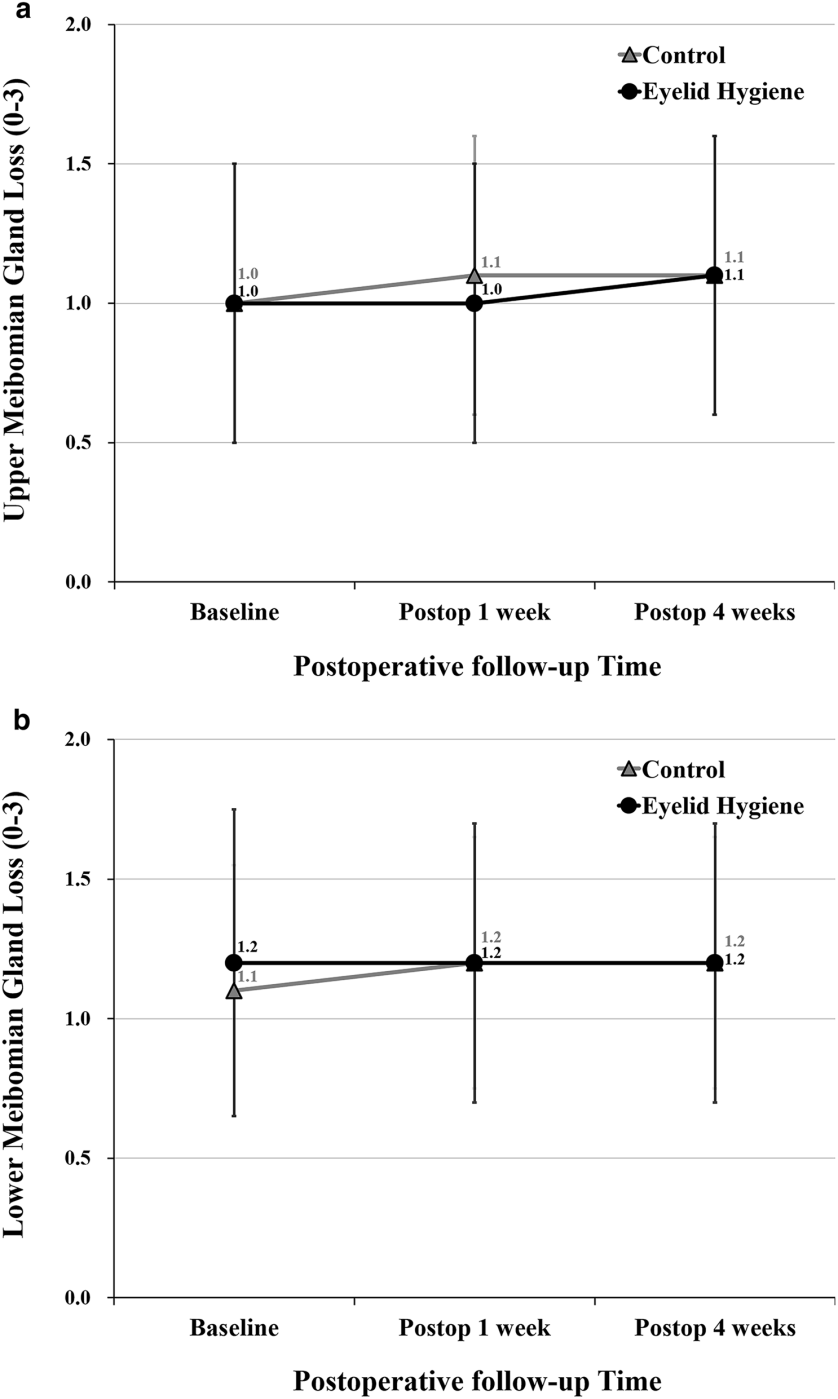
Comparison of meibomian gland loss grade in upper and lower eyelids between control and eyelid hygiene groups. (**A**) Upper meibomian gland loss. (**B**) Lower meibomian gland loss. Figures were created by Microsoft PowerPoint and Excel 2016.

The control group had significant worsening in meibomian gland expressibility in both the upper and lower eyelids (from 5.8 ± 2.0 to 4.8 ± 2.3 and 4.8 ± 2.2, respectively, in the upper eyelid; and from 4.4 ± 2.4 to 3.1 ± 2.6 and 3.3 ± 2.4, respectively, in the lower eyelid) at both 1 and 4 weeks postoperatively but the eyelid hygiene group did not (Table 2). The control group also had significant worsening in the meibomian gland secretion score in both upper and lower eyelids (from 1.0 ± 0.8 to 1.5 ± 1.0 and 1.5 ± 1.0, respectively, in the upper eyelid; and from 1.3 ± 1.0 to 1.7 ± 1.1 and 1.7 ± 1.0, respectively, in the lower eyelid) at both 1 and 4 weeks postoperatively but the eyelid hygiene group did not (Table 3). There were no significant changes in meibomian gland dropout in both the upper and lower eyelids during the 4 week period after cataract surgery in both eyelid hygiene and control groups (Fig. 4).

**Table 2:** Comparison of meibomian gland expressibility between control and eyelid hygiene groups.

	Control (n = 36) (Mean ± SD)	Eyelid Hygiene (n = 33) (Mean ± SD)	*P* value*
Upper gland expressibility (n/8)			
Baseline	5.8 ± 2.0	5.2 ± 2.7	0.339
Postop 1 week	4.8 ± 2.3	6.1 ± 2.1	**0.024**
Postop 4 weeks	4.8 ± 2.2	5.8 ± 2.2	0.053
*P* value*			
Baseline vs. postop 1 week	**0.015**	0.072	
Baseline vs. postop 4 weeks	**0.004**	0.222	
Postop 1 week vs. 4 weeks	> 0.999	0.496	
Lower gland expressibility (n/8)			
Baseline	4.4 ± 2.4	3.8 ± 2.9	0.374
Postop 1 week	3.1 ± 2.6	4.5 ± 2.7	**0.036**
Postop 4 weeks	3.3 ± 2.4	4.4 ± 2.7	0.078
*P* value*			
Baseline vs. postop 1 week	** < 0.001**	0.058	
Baseline vs. postop 4 weeks	**0.014**	0.273	
Postop 1 week vs. 4 weeks	> 0.999	> 0.999	

*SD* standard deviation.

* Repeated measures ANOVA with Bonferroni correction.

**Table 3:** Comparison of meibomian gland secretion score between control and eyelid hygiene groups.

	Control (n = 36) (Mean ± SD)	Eyelid Hygiene (n = 33) (Mean ± SD)	*P* value*
Upper gland secretion score (0–3)			
Baseline	1.0 ± 0.8	1.2 ± 1.0	0.485
Postop 1 week	1.5 ± 1.0	1.1 ± 0.9	0.071
Postop 4 weeks	1.5 ± 1.0	1.2 ± 1.0	0.154
* P* value*			
Baseline vs. postop 1 week	** < 0.001**	> 0.999	
Baseline vs. postop 4 weeks	** < 0.001**	> 0.999	
Postop 1 week vs. 4 weeks	> 0.999	> 0.999	
Lower gland secretion score (0–3)			
Baseline	1.3 ± 1.0	1.4 ± 1.1	0.936
Postop 1 week	1.7 ± 1.1	1.5 ± 1.2	0.457
Postop 4 weeks	1.7 ± 1.0	1.4 ± 1.1	0.240
*P* value*			
Baseline vs. postop 1 week	**0.018**	> 0.999	
Baseline vs. postop 4 weeks	**0.004**	> 0.999	
Postop 1 week vs. 4 weeks	> 0.999	> 0.999	

*SD* standard deviation.

*Repeated measures ANOVA with Bonferroni correction.

Lid hygiene compliance, measured by the percentage of eyelid cleansing conducted, was 90.0%. There were no complications associated with the use of Blephaclean, such as redness, swelling, or itchy eyelids, during the study period.

## Discussion

Cataract surgery can worsen blepharitis and meibomian gland function during the postoperative period to result in increased ocular discomfort and decreased vision and patient satisfaction^9–13^. Previous studies have shown that cataract surgery worsens eyelid margin abnormalities and meibomian gland function, including meibum quality and quantity^9,10^. Consistent with the results of previous studies, anterior blepharitis and meibum quality and quantity were worsened after cataract surgery in the control group in this study. Anterior blepharitis and MGD worsen after cataract surgery for several reasons. First, all patients are advised not to get water in their eyes and not to rub their eyes after cataract surgery, while the recommended duration varies from hospital to hospital. Inability to wash the face and touch the eyes may contribute to poor eyelid hygiene that may worsen anterior blepharitis and MGD. Second, subbasal corneal nerve fibers are reduced and corneal sensitivity is decreased after clear corneal incision cataract surgery^24,25^. Recovery of subbasal corneal nerve fibers takes about 8 months, and recovery of corneal sensation takes about 1 to 3 months^24–26^. Decreased corneal sensitivity causes decreased blinking rate^27^ and incomplete blinking, which might contribute to MGD progression because meibum is delivered from meibomian glands by squeezing of the orbicularis oculi and Riolan muscles^28–31^. Third, the use of an eyelid speculum may induce eyelid dysfunction in early postoperative periods^32,33^ to result in inappropriate blinking and squeezing force to deliver meibum from meibomian glands^9,12^. Fourth, increased ocular surface inflammation after cataract surgery may influence eyelid margins and meibomian gland function^10,12^.

We hypothesized that one of the most important factors for worsening of anterior blepharitis and MGD after cataract surgery is poor eyelid hygiene in the operated eye. Thus, we wanted to determine whether eyelid hygiene before and after cataract surgery is effective at preventing postoperative anterior blepharitis and MGD deterioration. The results of this study demonstrate that perioperative eyelid hygiene prevented exacerbation of anterior blepharitis and meibum quality and quantity. In the control group, worsening of the grade of eyelid debris and telangiectasia at 1 week after surgery was not prolonged until 4 weeks after cataract surgery without additional eyelid hygiene. In this study, patients were advised not to wash their face for 5 days after cataract surgery. Therefore, patients were able to wash their faces at six days after surgery, and eyelid hygiene may have improved. As a result, anterior blepharitis may have improved at 4 weeks postoperatively. Worsening of meibomian gland expressiblity and secreted meibum quality persisted for 4 weeks postoperatively in the control group. Previous studies have shown that altered meibomian gland function persisted for 3 months after cataract surgery^9,34^. Eyelid hygiene before and after cataract surgery is important because deterioration of meibum quality and quantity after cataract surgery can be prevented through eyelid hygiene.

ASCRS Cornea Clinical Committee developed a consensus-based algorithm for diagnosing and treating ocular surface diseases including MGD before refractive and cataract surgery^13^. This algorithm highlights the importance of meibomian gland expression and meibography to identify MGD and treatment of anterior blepharitis and MGD by warm compress and eyelid hygiene^13^. Warm compress methods used at home do not allow the eyelids to reach the proper treatment temperature, and it is burdensome to directly contact eyes with a warm compress device that is non-sterile in the early postoperative periods. Conversely, preservative-free disposable sterile eyelid wipes, such as Blephaclean used in this study, are considered safe to use immediately after intraocular surgery. No complications associated with postoperative use of Blephaclean were observed in this study. Blepharitis is a major risk factor for endophthalmitis after cataract surgery^35^, and eyelid hygiene before ophthalmic surgery significantly reduces microbial flora in the eyelid and conjunctiva, the sources of infecting organisms^23^. Perioperative eyelid hygiene is recommended for patients undergoing intraocular surgery to prevent anterior blepharitis and MGD deterioration as well as postoperative infection.

A previous study showed that there was no change in tear volume measured using Fourier domain-OCT before and after cataract surgery, although ocular discomfort increased after cataract surgery^9^. Thus, aqueous tear volume itself does not explain the increase in ocular discomfort after cataract surgery^9^. In addition, Park et al,. showed that topical antibiotics and steroid use did not improve the worsening of eyelid margin abnormalities and meibum qualitative/quantitative changes in patients with dry eye after cataract surgery^12^. Subjective symptoms, TBUT, and ocular surface staining have been reported differently depending on the study. After cataract surgery, these parameters were worsened in some studies and improved or remained similar in other studies^9,11,12,34^. The differences in postoperative changes of subjective symptoms, TBUT, and ocular surface staining in each study are thought to be due to differences in preoperative dry eye syndrome and MGD status among studies and postoperative use of topical antibiotics and steroids^9,11,12,34^. Previous studies used topical 0.5% levofloxacin or 0.5% moxifloxacin and 1% Prednisolone acetate or 0.1% dexamethasone but not topical NSAIDs postoperatively, while this study used topical 0.1% bromfenac sodium twice daily in addition to the topical antibiotic and steroid. Similar to previous results, there were no changes in subjective symptoms, TBUT, and ocular surface staining after cataract surgery in the control group. However, eyelid hygiene before/after cataract surgery improved postoperative subjective symptoms in the eyelid hygiene group, and TBUT was higher in the eyelid hygiene group than in the control group at 4 weeks postoperatively. In addition, anterior blepharitis and meibomian gland function were significantly worsened after cataract surgery in the control group, although all patients used topical antibiotic, steroid, and NSAIDs for 1 month from 3 days before to 4 weeks after cataract surgery. Therefore, it is believed that additional eyelid hygiene after cataract surgery is necessary for patients undergoing cataract surgery.

There are a number of limitations to this study. First, the sample size is relatively small. A larger, randomized controlled clinical trial is needed to validate the efficacy of eyelid hygiene before/after cataract surgery in preventing the development and worsening of blepharitis and MGD. Second, in this study, the subjects were not masked to the group assignments. Although inevitable, this single-masked design might be a source of bias.

## Conclusion

Lid hygiene before/after cataract surgery improved postoperative subjective symptoms and prevented postoperative exacerbation of anterior blepharitis, meibomian gland expressibility, and meibomian gland secretion score. Thus, perioperative eyelid hygiene using preservative-free disposable sterile eyelid wipes is needed to improve subjective symptoms and prevent blepharitis and MGD exacerbation after cataract surgery.

## Methods

This multicenter, investigator-masked, randomized controlled parallel-group study was conducted between July 2018 and November 2019 at eight institutions (Korean Meibomian Gland & Ocular Surface Study Group) in South Korea^36^. This study followed the tenets of the Declaration of Helsinki and Good Clinical Practice. The prospective study protocol was approved by the Institutional Review Boards of Korea University Ansan Hospital (2018AS0032) and each institution, and the study was registered as a clinical trial at https://cris.nih.go.kr (registration number: KCT0002825, registration date: 04/24/2018) and conformed to the CONSORT checklist. After explaining the purpose of the study and possible results, all patients provided signed informed consent to participate in this study.

### Study population

This study enrolled subjects diagnosed with cataract and obstructive MGD who wanted to undergo cataract surgery. Obstructive MGD was diagnosed when one or more of the following signs were observed: (1) altered meibomian gland secretion upon gentle expression using a cotton swab such as absent, viscous, or waxy white secretion; (2) presence of two or more telangiectasias at eyelid margin; (3) pouting and/or plugging of two or more meibomian gland orifices^36,37^. Exclusion criteria included (1) previous ocular damage or active ocular infection; (2) presence of any uncontrolled systemic diseases; (3) use of contact lens within one month of inclusion in the study; (4) allergy to fluorescein sodium or topical anesthetic; (5) history of ocular surgery within six months of inclusion in the study; (6) systemic drug user (tetracycline derivatives, antihistamine, isotretinoin); (7) eyelid disease or eyelid structural abnormality other than blepharitis and MGD; and (8) aged less than 20 years old^36^.

### Study protocol

Subjects were randomly assigned in 1:1 ratio to one of two groups of eyelid hygiene group and control group by independent clinical research coordinator according to a computer-generated randomization list. Randomization sequence was created between 1 and 2 using the RAND function in Excel 2013 (Micrsoft Inc., Redmond, WA) and was stratified with a 1:1 allocation. In the eyelid hygiene group, eyelid hygiene was performed in the morning and evening twice a day using a Blephaclean (Laboratoires Théa, Clermont-Ferrant, France) for 10 days, from 3 days before to 1 week after cataract surgery (total of 20 eyelid cleansing sessions were recommended). In the control group, eyelid hygiene was not performed before or after cataract surgery. All participants completed a patient history and subjective symptom questionnaire. Afterward, an ophthalmic examination with slit-lamp biomicroscopy and clinical measurements were performed in the following order to observe the eyelid margin, ocular surface, and meibomian glands to ensure that previous examinations did not impact subsequent examination: (1) corneal and conjunctival surface staining using fluorescein; (2) tear film break-up time (TBUT); (3) anterior blepharitis (lid debris, eyelid redness/swelling, and telangiectasia); (4) meibomian gland expressibility and secretion score; and (5) meibography^3^.

### Cataract surgery

All phacoemulsification and intraocular lens (IOL) implantation were performed by one experienced surgeon in each institute. A 2.2–2.75 mm clear corneal incision was made after topical anesthesia with proparacaine hydrochloride 0.5%. Continuous curvilinear capsulorrhexis (CCC) was created with a 26 gauge needle and CCC forceps. Standard phacoemulsification technique was used, and the IOL was inserted into the capsular bag. Preoperatively, all patients were prescribed topical levofloxacin (Cravit; Santen Pharmaceutical, Osaka, Japan) and 0.1% fluorometholone 4 times daily, and 0.1% bromfenac sodium (Bronuck; Taejoon Pharm, Seoul, Korea) twice daily for 1 month from 3 days before to 4 weeks after cataract surgery. All patients were instructed to avoid getting water in the operated eyes and to not rub their eyes for 5 days after surgery.

### Subject examination

All subjects completed the SPEED subjective symptom questionnaire (0–28) and underwent ophthalmic examination by a masked examiner to evaluate objective signs at baseline and at postoperative 1 and 4 weeks. Corneal and conjunctival surface staining grade with fluorescein sodium-impregnated paper strips (Haag-Sterit, Bern, Switzerland) was evaluated according to the Oxford scoring scheme (0–5)^38,39^. TBUT was measured three times using a stopwatch, and the mean value of the three measurements was recorded to one decimal place^36^.

To evaluate degree of anterior blepharitis, both upper and lower eyelids were observed with slit-lamp biomicroscopy. eyelid debris and eyelid redness/swelling was graded from 0 to 4 (absent, mild, moderate, severe, and very severe)^40,41^. Eyelid telangiectasia was graded from 0 to 3 (no findings, mild, moderate, and severe)^42^. A standard set of color photographs for each grade of eyelid debris, eyelid redness/swelling, and telangiectasia was made from previous articles and provided to each institution to unify evaluation of anterior blepharitis^41,42^.

To identify meibomian gland expressibility and secretion score, both upper and lower eyelids were gently pressed using a cotton swab. The number of expressible glands among the central eight glands was counted (0–8) and meibomian gland secretion quality of the central eight glands was graded from 0 to 3 (clear fluid, cloudy fluid, cloudy particulate fluid, and inspissated, like toothpaste)^43,44^. The degree of meibomian gland drop out in the middle two-thirds of the eyelid was graded from 0 to 3 (area of MG loss between 0 and 25%, 26–50%, 51–75%, and 76–100%)^43,45^. To evaluate meibomian gland expressibility, secretion score, and drop out, each upper and lower eyelid was separately evaluated. All examinations were performed by a masked investigator at each institution. The percentage of eyelid cleansing conducted by subjects was evaluated at postoperative 1 week to evaluate eyelid hygiene compliance in the eyelid hygiene group.

The primary endpoint with respect to efficacy of eyelid hygiene in blepharitis and MGD after cataract surgery was the changes in SPEED score, degree of anterior blepharitis, and meibum quality and quantity from baseline to 1 and 4 weeks after cataract surgery. The secondary endpoint was the changes in ocular surface staining and TBUT from baseline to 1 and 4 weeks after cataract surgery.

### Statistical analysis

Statistical analyses were performed with IBM SPSS Statistics Standard 20 (IBM Corp., Armonk, NY, USA). In the previous study, the mean preoperative symptom score was 11.21 and the standard deviation (SD) was 9.49 in patients with MGD. The symptom score increased to 19.59 ± 10.99 after 1 month of cataract surgery^10^. The null hypothesis was that eyelid hygiene can improve patient symptoms at the same level as before surgery. Based on these results, 33 subjects were needed to achieve a power of 90% and a 5% significance (2-tailed) to identify the difference in means. Due to the nature of prospective study, we decided to register the number of subjects in the experimental group as 40, taking into account the follow-up loss rate (20%). For the pilot study comparing 1: 1 with the experimental group, the number of subjects in the control group was set as 40. The GPOWER 3.1 program was used to calculate the number of subjects^46^. Student’s t-tests, Fisher’s exact tests, and repeated measures ANOVA with a Bonferroni correction were performed to compare the clinical characteristics and measurement results between the eyelid hygiene and control groups. Values were expressed as means and standard deviations. A *P*-value of less than 0.05 was considered statistically significant.
